# Barriers and Facilitators to Eye Donation in Hospice and Palliative Care Settings: A Scoping Review

**DOI:** 10.1089/pmr.2021.0017

**Published:** 2021-06-16

**Authors:** Banyana Cecilia Madi-Segwagwe, Mike Bracher, Michelle Myall, Tracy Long-Sutehall

**Affiliations:** School of Health Sciences, University of Southampton, Southampton, United Kingdom.

**Keywords:** end-of-life care, eye donation, palliative care, perception, practice, scoping review

## Abstract

***Background:*** The need for eye tissue for use in sight saving and sight restoring surgery is a global issue. Approximately 53% of the world's population has no access to interventions such as corneal transplantation. Low levels of eye tissue impact on service providers such as National Health Service Blood and Transplant who aim to achieve a weekly stock of 350 eyes but do not meet this target.

***Aim:*** Patients who die in hospice and palliative care settings could be potential donors; therefore the aim of this systematic scoping review was to identify the potential for eye donation and barriers toward it from these clinical contexts.

***Design:*** A scoping review following the Joanna Briggs scoping review methodology was applied to search the global literature.

***Results:*** 13 articles from the global literature were retrieved. Evidence indicate that 542 patients could potentially have donated their eyes. Key barriers to increasing eye donation include the reluctance of healthcare professionals to raise the option of eye donation and the evidenced lack of awareness of patients and family members about donation options and eligibility. This review also indicates a lack of clinical guidance drawn from high-quality evidence proposing interventions that could inform clinical practice and service development.

***Conclusion:*** The scoping review presented here provides an up-to-date view of the current potential for, perceptions toward, and practice underpinning offering the option of eye donation to dying patients and their family members in hospice and palliative care context.

## Introduction

The need for eye tissue is a global issue. Gain et al.^1^ indicate that ∼53% of the world's population has no access to interventions such as corneal transplantation reporting that globally, only one cornea is available for the 70 that are needed. These data highlight an ongoing disparity in supply and demand for eye tissue in most countries worldwide. Furthermore, >2 million people in the United Kingdom are living with sight loss, which is predicted to increase to 4 million by 2050.^2^ Corneal blindness is the fourth leading cause of blindness worldwide with an estimated 80% of all cases being avoidable and reversible.^3,4^

## Evidenced Barriers to Increasing Eye Donation

International empirical data report that low levels of eye donation outside of Intensive Care Units and Emergency Departments^5–8^ is due to negative attitudes toward eye donation held by health care providers (HCPs),^9–13^ negative public views regarding eye donation,^14,15^ and low levels of support on the Organ Donor Register (ODR).^16^ Recent data indicate that 85% of registrants on the ODR indicated a willingness to donate all organs and tissues but of those who log a restriction, 68% decline eye donation.^16^ Furthermore, recent data from U.K. Hospice Care settings^17,18^ identified that the majority of staff had rarely or never raised the topic of eye donation with patients or relatives as part of end-of-life care planning.

Low levels of eye tissue have a direct impact on service providers such as NHS BT who aims to achieve a weekly stock of 350 eyes for use in transplant and other sight saving surgery, but current stocks are ∼150 eyes per week (personal communication with tissue services April 12, 2020). There is a need to achieve a sustained supply of eye tissue and as patients who die in palliative and hospice care settings could be potential eye donors,^2,19^ this article presents a scoping review of the global literature that specifically looks at the barriers and facilitators to achieving eye donation from these settings.

## Review Methodology

**FIG. 1.**
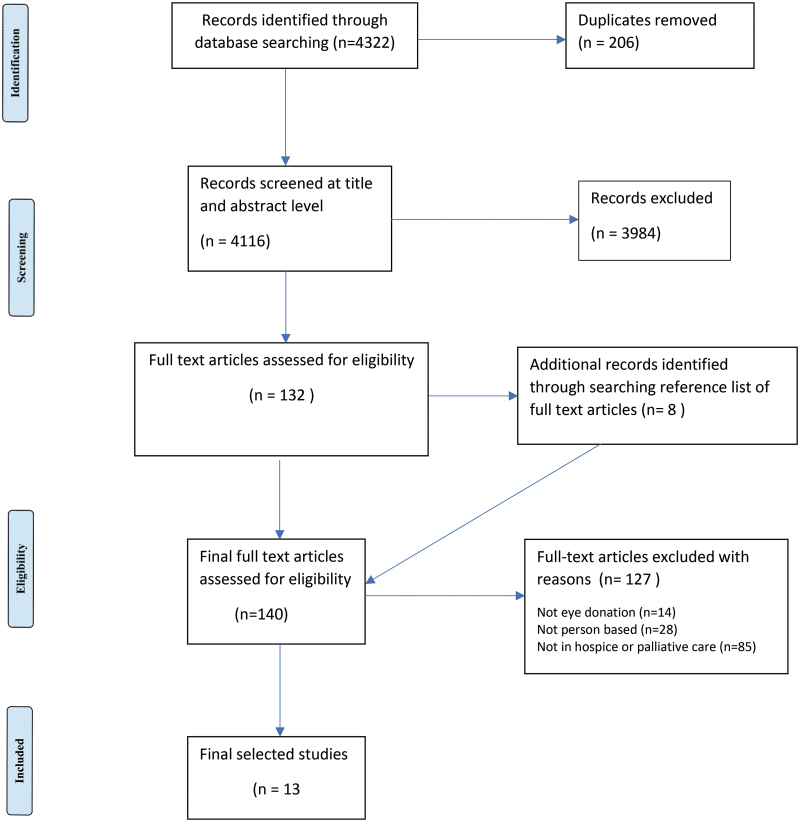
Process followed in the selection of included studies using the Preferred Reporting Items for Systematic Reviews and Meta-Analyses.^21^

This scoping review followed the Joanna Briggs Institute (JBI) framework for scoping review^20^ (Table 1) and used the Preferred Reporting Items for Systematic reviews and Meta-Analyses extension for Scoping Reviews (PRISMA-ScR) checklist to illustrate selection of the final included articles (Fig. 1).^21^ Scoping review methodology was deemed appropriate to identify the scope, coverage, and type of research currently available on a topic, map the available evidence, and generate a synthesis of the available knowledge.^22–24^

**Table 1.** Joanna Briggs Institute Framework for Scoping Review^20^

1	Defining and aligning the objectives and questions
2	Developing and aligning the inclusion criteria with the objectives and questions
3	Describing the planned approach to evidence searching and selection
4	Searching for the evidence
5	Selecting the evidence
6	Extracting the evidence
7	Charting the evidence
8	Summarizing the evidence in relation to the objectives and questions
9	Consultation of information scientists, librarians, and/or experts throughout

## Review Question and Objectives

### Review question

What are the evidenced barriers and facilitators to eye donation in Hospice and Palliative care settings?

### Objective 1

To systematically map the current international evidence base relating to eye donation in hospice and palliative care settings.

### Objective 2

To identify the factors that are evidenced as informing or influencing the option of eye donation being discussed with service users in hospice and palliative care settings.

## Inclusion Criteria

The inclusion/exclusion criteria were developed in line with the JBI framework clarifying the Population (P) Concept (C) and clinical Context (C) (PCC), type of evidence sought, and other limiters within which the search was bounded (Table 2).

**Table 2.** Inclusion/Exclusion Criteria

	Inclusion	Exclusion
Population	Patients (*donor, deceased donor, and potential donor*) Carers (*relative, next of kin, family member, and informal carer*) Members of the public Healthcare professionals (*physician, doctor, and nurse*)	Children, young people, and adolescents
Concept	Barriers and facilitators to eye tissue donation (*perceptions, preferences, practice, potential, views, attitudes, beliefs, experience, and knowledge*)	Organ, body, egg, sperm donation, and surgery to the eye
Context	Hospice and palliative care settings	Acute care areas, for example, intensive care units, critical care units, emergency departments, eye banks, process of retrieval, storage, and treatment of eye tissue
Type of study	Empirical research, clinical guidelines, expert opinion, letters to editors, initial reporting of findings, and literature reviews	
Language	Full article in English language	Non-English language
Year of publication	1983–2020	

## Evidence Searching and Selection

An initial search was undertaken in the PubMed database using the terms “Eye[MeSH] AND Tissue Donation[MeSH]” (Table 3). The search was limited to articles published during or after the establishment of the U.K. Corneal Transplant service.^25^ However, the resulting 190 articles did not include several key articles known to the review team. Therefore, 23 articles (the development set) known to the review team and verified against the inclusion criteria were compiled and checked for indexing in PubMed.

**Table 3.** Final Search Strategy and Results by Database (All Searches Conducted January 27, 2020)

Search terms		
(Eye OR cornea^*^ OR ocular) AND (donat^*^ OR donor^*^) NOT “eye drop^*^” NOT acqueous NOT genetics NOT histology^*^ NOT membrane NOT microscopy NOT MRI NOT oculoplast^*^ NOT oocyte NOT endotheli^*^ NOT keratoplat^*^		
Database	Results	Limits applied
PubMed	3602	Species: Humans
CINAHL	141	Exclude MEDLINE records
Embase Classic+Embase	288	Exclude MEDLINE records
PsychInfo	186	None
Epistemonikos	34	No PMC (PubMed)
Cochrane reviews	71	None
Total retrieved	4322	

Results showed that only five (28%) could be retrieved in PubMed. Seventeen of the 18 excluded articles were not indexed under the term “Eye[MeSH] despite six of them having eye related terms in the title and/or abstract.” MeSH or equivalent database-specific terms were dropped for further searchers. Screening of title, abstract, and full article of the 190 articles from the initial search resulted in 70 articles being added to the developmental set of 23 articles previously identified, producing a test set *n* = 93 records. Finally, a two-stage process aiming to maximize the sensitivity of the search strategy and minimize the number of irrelevant records (specificity) was implemented.^26^

Stage 1 applying search terms—“(eye OR cornea*) AND (donat* OR donor*)” returned 10,313 records [retrieving 85 (91.4%) of the test set]. Stage 2 involved iterative stepwise identification and testing of exclusion terms (specified by the Boolean term “NOT”) to exclude irrelevant records while maintaining the 91.4% level of sensitivity. The team used the PubMed PubReMiner tool^27^ to identify potential exclusion terms (PubMed PubReMiner allows users to see frequency tables of occurrences of relevant terms from articles included in a given search, and their associations with other attributes such as topics or keywords).^27^

We consulted specialist subject librarians at University of Southampton throughout development of the search terms.^28^ This strategy was repeated across five additional databases returning a total of 4322 records from all sources (Table 3).

## Selecting the Evidence

The final screening process following the PRISMA-ScR framework for reporting scoping reviews^21^ is illustrated in Figure 1. After removing duplicates (*n* = 206) from the 4322 hits, 4116 records were exported to Microsoft Excel for title and abstract review by authors (B.C.M.-S., M.B., and T.L.-S.). After title and abstract review, 3984 articles were excluded resulting in 132 articles for full review. The reference lists of the 132 articles were searched resulting in 8 additional articles being included. Full review of the 140 articles was undertaken by two authors (B.C.M.-S. and M.B.) with any disagreements resolved by T.L.-S.

One hundred twenty-seven articles (of 140) were excluded: 14 records did not relate to eye donation (i.e., these contained only incidental references to eye donation, or did not include a significant focus on it); 28 did not relate to “perceptions, preferences, views, attitudes, beliefs, experience, knowledge” elements of our PCC, whereas 85 were not in the context of hospice and palliative care (as relevant articles often did not clarify this context in the title, abstract, or keywords). Thirteen records met the inclusion criteria and were included in the final review.

## Extracting the Evidence

Authors, aims/purpose; study design/methodology, participants/sample size, method of data collection and analysis, finding and limitations were extracted from the final 13 records and reported in Table 4. Studies listed in Table 4 are referenced in the text by numerals in square brackets.

**Table 4.** Data Extraction Summary

Study ID	Study references Country, context	Aims/purpose	Study design, participants	Data collection and analysis methods	Findings
1	Wells J, Sque M: Living choice: The commitment to tissue donation in palliative care. Int J Palliat Nurs 2002;8:22–27. United Kingdom, Palliative care setting	To explore how nurses and doctors feel about tissue donation in palliative care	Grounded theory study Healthcare Professionals (HCP) (*n* = 8)	Data collection: Semistructured interviews Data analysis: Grounded theory analysis	Patients in palliative care should have the opportunity to be consulted about their wishes and expect to be informed and consulted about tissue donation. However, in units where donation was routinely discussed, participants generally consulted relatives rather than patients resulting in concerns that patients were not involved in decision making. Patients or family members “openness” to discussing end-of-life planning led to HCPs being more comfortable raising the option of donation. Discussing donation should be a multiprofessional role. Timing of the discussion is crucial to the outcome. A main concern was whether the request for donation and the donation process would cause the patients and families any physical or psychological harm.
2	Carey I, Forbes K: The experience of donor families in the hospice. Palliat Med 2003;17:241–247. United Kingdom, Hospice care setting	To evaluate the experiences, attitudes and feelings of relatives who had consented to the donation of corneas of a loved one within a palliative setting	Semistructured interviews with 12 family members (carers/relatives)	Data collection: Semistructured interviews Data analysis: Framework analysis	*Awareness of eligibility* Almost all participants were not aware that their deceased relative was eligible for donation and reported that they would not have reached the decision to donate on their own. Participants stated that they would have been distressed if they had not been given the option to donate and later realized, they could. *Raising the topic of eye donation:* discussions were initiated by nurses generally after the death of the patient. The timing and approach were acceptable to families although they felt it would have been easier if they had known beforehand. *Participants views of eye donation:* Most participants felt that donation was right and had a positive experience with the process and felt they had done something worthwhile. Social policy: 10 participants stated that the patient should make the decision about donation. All participants felt that there should be publicity and discussion about organ donation preferably before the person was faced with incurable disease or imminent death.
3	Gillon S, Hurlow A, Rayment C, et al.: Obstacles to corneal donation amongst hospice inpatients: A questionnaire survey of multidisciplinary team member's attitudes, knowledge, practice and experience. Palliat Med 2011;26:939–946. United Kingdom, Hospice care setting	To explore the attitudes, knowledge, practice, and experience of corneal donation from hospice staff with direct clinical contact with patients	Survey shared with 704 clinical multidisciplinary team members in 12 hospices	Data collection: paper questionnaire with fixed response and free text option Data analysis: descriptive statistics and thematic analysis of free text comments	70% (*n* = 291/418) of respondents perceived corneal donation to be a rewarding opportunity for patients and/or their families. 88% (*n* = 375/425) stated it was important that patients knew that they could donate. 43% (*n* = 118/427) (43%) indicated that corneal donation should be discussed routinely with eligible patients. 17% (*n* = 72/418) felt that discussing corneal donation would be too distressing for a patient and/or their family. 37% (*n* = 156/422) were not sure whether they were comfortable enough to start a conversation about corneal donation with a patient or a family member. 34% felt that it was part of their role and 39% (*n* = 161/421) felt that it was someone else's role to raise the issue of corneal donation with patients and/or their family members. 93% (*n* = 399/431) rarely or never raised the option of eye donation. Key reasons for not engaging in discussions were: Concerns about the impact of the discussion on patients and families A belief HCPs lacked essential knowledge, about the process of eye donation. A perception that donation is not part of hospice culture.
4	Kuo S, Chou P, Liao Y, et al.: Perspectives of decision-making for corneal donation: A qualitative research among cancer patients. OMEGA J Death Dying 2018;0:1–8. Taiwan, Palliative care setting	To identify the views of terminal cancer patients toward corneal donation	Exploratory qualitative study with 25 cancer patients	Data collection: Semistructured interviews Data analysis: content analysis	*Key findings were that:* Participants felt that the issue required family members to indicate their preferences. Participants preferred to maintain their bodies intact because of the deeply held beliefs that the body must remain intact after death. Participants believed that corneal donation was against their Buddhist religious beliefs as they believe that the body should be untouched for eight hours after death. Participants also believe that spirit should be able to see and, therefore, eyes should not be removed.
5	Walker L, Neoh K, Gilkes H, Rayment C: A qualitative study using semistructured interviews of palliative care patients' views on corneal donation and the timing of its discussion. Palliat Med 2018;32:1428–1437. United Kingdom, Palliative care setting	To understand views and feelings of patients in palliative care settings toward corneal donation.	Exploratory qualitative study with 9 patients	Data collection: Semistructured interviews Data analysis: Thematic analysis	Patients' baseline knowledge was very limited and most did not know anything about corneal donation before the study. Altruism was a key influence, patients felt positive about being able to help someone else. Eyes were not perceived as being different to other organs and participants valued sight and felt it would be important to help someone see again. All participants acknowledged the role played by their family in decision making and were keen to involve them. Participants felt they would prefer to talk about donation when they were well rather than when vulnerable and close to death. Participants felt discussions about donation was a covert way to tell someone that they were dying. Participants were open to discussing donation with healthcare professionals and felt it would be easier with someone they already had relationship with. Although participants said they did not know anything about donation, they, however, felt they would not be eligible to donate. Some thought they could pass on their cancer if they donated.
6	Ng I, Astle J, Tregenna E, et al.: Health services and policy. Future Healthcare J 2019;6:s38. United Kingdom, Hospice care setting	To assess factors that influence corneal donation within the palliative care service	Survey of 37 HCPs and 11 patients Retrospective note review of 84 deceased patient records 2016	Data collection: Retrospective note review Questionnaire (developed by Gillon et al.) distributed to healthcare providers in 2014. Questionnaire to patients admitted to the service between June and August 2015. Data analysis: descriptive statistics.	*Results of retrospective note review:* 85 deceased patients' notes were reviewed against eye donation criteria. Of these 35% (*n* = 30) were judged to be eligible for corneal donation with a further 11% (*n* = 10 patients) potentially eligible. *Results of survey healthcare professionals:* 92% (*n* = 92) of respondents never or rarely raised the subject of corneal donation with patients or relatives. 76% (*n* = 76) of respondents had not received any information or training regarding corneal donation. 81% (*n* = 81) of respondents felt they did not know enough about corneal donation to discuss it with patients or relatives. Knowledge and training were identified as significant barriers to raising these discussions. *Results of questionnaire with inpatients*: 6/11 (54.5%) participants had not heard of corneal donation and all were either glad or neutral about being informed about corneal donation. 8/11 (73%) participants did not find it upsetting to discuss corneal donation and the remaining 3/11 (27%) indicated although they found it upsetting, they would rather have a conversation than not. Patients' decisions about donation changed after discussions of eye donation. Before discussion none of the patients were planning to donate their corneas, whereas after the discussion 7/11 (64%) were planning to donate.
7	Niday P, Painter C, Peak J, et al.: Family and staff responses to a scripted introduction to tissue donation for hospice inpatients on admission. Prog Transplant 2007;17:289–294. USA, Hospice care setting	To implement and evaluate a change in practice to offer information about tissue donation as part of admission process	Service evaluation-written logs of 12 healthcare providers	Data collection review of nurses' logs	Comments on nurses' logs indicated no concerns from patients and families to receiving information about donation at admission. There was less frustration from families at the time of death and introduction of donation Nurses were more positive about introducing the option of donation at admission compared with when donation was introduced at the time of death. Nurses' logs confirmed that patients and families were not aware that they could donate. Corneal donation increased by 250% during a six-month period from 2 out of 32 eligible corneal donors to 7 out of 34 eligible donors.
8	Tredget K, Ward-Davis L: Responding to the public's voice: Changing cornea donation practice in a hospice (Letter to the editor). BMJ Support Palliat Care 2017;0:1–2. United Kingdom, Hospice care setting	Service evaluation of the introduction of personalized plan of care for dying patients that included the option for tissue and organ donation	Service evaluation reporting outcome of retrospective note review, staff survey of 14 HCPs. Telephone interviews with five family members	Data collection: questionnaire with HCPs Retrospective note review Telephone interviews with family members of deceased hospice inpatients Data analysis: Descriptive statistics	Findings from questionnaire with HCPs 12/14 (86%) of doctors felt that discussing eye donation did not cause additional distress to patients. 8/14 (57%) of doctors reported that the discussions had been helpful to patients and families as donation provided an opportunity to give something back and enabled a positive outcome from the death. *Note review:* On average 240 deaths occurred each year at this hospice. Before 2015 no patients had been referred for eye donation. 67/77 (87%) were eligible to donate. 34/67 (51%) of eligible patients' relatives were approached about donation before the patient's death. 15/34 (44%) of those invited to consider donation subsequently donated their corneas *Reasons for non-discussion of eye donation* Speed of deterioration, concern about exacerbating already significant distress and lack of clinician clarity on eligibility criteria were common reasons. *Reasons for declining donation* Previously expressed wish by patient not to donate or family's uncertainties about the patient's wishes were reasons for decline. Findings from telephone interviews: Interviews with deceased relatives indicated that relatives felt it was acceptable to raise the option of donation and felt that it did not add to their distress.
9	Roach R, Broadbent AM: Eye donation in Sydney metropolitan palliative care (Letter to the editor). J Palliat Med 2009;13:121–123. Australia, Palliative care setting	To identify factors contributing to low rate of eye donation from palliative care unit in Sydney metropolitan area	Retrospective audit of 2000 deceased patient records	Data collection: Retrospective note review Data analysis: Descriptive statistics	2000 deceased patients' notes were reviewed over a one-year period. 50 (2.5%) patients became eye donors. Donors came from only four out of the nine palliative care units (44%) Two (22%) units provided 90% (*n* = 45) of the eye donations. Palliative care units do not appear to discuss or promote eye donation with patients or their families.
10	Gillon S, Hurlow A, Rayment C, et al.: Eligibility for corneal donation within the hospice population (Letter to the editor). Palliat Med 2010;24:551–552. United Kingdom, Hospice care setting	To quantify percentage of inpatients eligible to donate corneas and number with whom donation is discussed	Observational retrospective note review of 100 deceased patient records (September–December 2008)	Data collection: patient note review Date analysis: Descriptive statistics	100 deceased patients' notes were reviewed. There were no contraindications to eye donation for 52 patients (52%), whereas 15 (15%) had definite contraindications. No documentation regarding discussion of corneal donation was recorded.
11	Stiel S, Hermel M, Radbruch L: Cornea donation from patients deceased at a palliative care unit (Letter to the editor). Palliat Med 2010;25:183–184. Germany, Palliative care setting	To assess the potential for corneal donation and the relative rate of actual donation	Observational retrospective note review of 704 deceased patient records	Data collection: Review of patient electronic records Data analysis: Descriptive statistics	704 deceased patients' notes were reviewed between 2003 and 2009. 229/704 (32.5%) patients were potential donors 112/704 (49%) patients gave consent for cornea donation
12	Edwards P: Corneal donation within palliative care: A review of the literature. Int J Palliat Nurs 2005;11:481–486. United Kingdom, Palliative care setting	To examine whether the option of donation is being offered in a systematic manner To explore the moral dilemmas involved in corneal donation and the implications for nursing practice and research	Literature review in Medline and Cinhal databases	Data collection: Review of studies covering period 1995–2005 Data analysis Thematic analysis	Findings from literature review *Corneal donation is rarely offered.* Cumulative findings indicated that family members were surprised that their relative could donate. Family members would have been distressed if they had not been offered the option to donate and found out later that this could have been an option; low numbers of family members raise the issue of eye donation. Families felt that donation did not have any effect on their bereavement. Family members desire to fulfil deceased wishes, give meaning to the death and the families' own views about donation influenced donation decisions. Healthcare professionals often do not raise the issue of corneal donation, often they just respond to requests by patients or family members.
13	Spencer M: The barriers to organ and tissue donation in palliative care. End Life J 2012;2:1–11. United Kingdom, Palliative care setting	To explore the involvement of palliative care patients in decisions about donating their tissues To explore why families may be reluctant to consent to donating organs/tissues of deceased loved ones To explore why nurses are wary of discussing the possibility of donation with patients and/or their next of kin	Literature review in British nursing index, CIHAHL, MEDLINE, Embase and PsycINFO	Data collection: Review of the literature with no date limits Data analysis Thematic analysis	Findings from literature review There is no consensus among palliative healthcare professionals about whether and/or when patients should be involved in discussions about donation. Patients often spontaneously discuss end-of-life matters such as their funeral arrangements, their will, and expectations for the time they have left, but they rarely spontaneously discuss organ or tissue donation. Introducing donation discussions increased donation rates. Patients should be given appropriate information to enable them to make choices about donation. Knowing the wishes of the deceased regarding donation influences next of kin donation decision making. Healthcare professionals find it challenging to talk about donation even though they frequently have difficult discussions about death and dying. HCPs are fearful of the reaction of patients and families to the request for donation. The attitudes of healthcare providers influence their practice, those with negative views and less knowledge about donation are less likely to discuss it compared with those who are positive and have more knowledge.

HCP, health care provider.

## Charting the Evidence

Thirteen records that met the inclusion criteria were exported to Atlas.ti 8^29^ for management and analysis. Articles were analyzed in line with the review objectives.

### Objective 1: To systematically map the current evidence base relating to eye donation in hospice and palliative care settings

#### Year of publication

The search date range was set as 1983 to 2020. This date range commenced from the date of setup of the first U.K. corneal transplant unit^25^ until March 2020. No publications were retrieved between the date range of 1983 and 2000. Eight publications were retrieved between 2001 and 2011 [1–3,7,9–12]. Five publications were retrieved between 2012 and 2020 [4–6,8,13]. Search results indicated that there has been an evidenced increase in publications linked to eye donation from palliative and hospice care settings since 2001. This may be in response to increasing awareness of the shortage of eye tissue for use in transplantation and medical research and the recognition of the potential for donation from these settings.

#### Countries and contexts

Retrieved publications included articles from five countries, including the United Kingdom (*n* = 9) (four were in palliative care context [1,5,12,13] and five in hospice care contexts [2,3,6,8,10]) and four countries generated one publication each in the United States (in a hospice setting) [7], Taiwan [4], Australia [9], and Germany [11] (all in palliative care contexts).

#### Methodology/design

Of the 13 retrieved publications, six reported empirical research [1–6], four of the empirical studies were qualitative studies [1,2,4,5], one was a survey [3], and one a mixed method study combining a survey and retrospective patient note review [6]. There was one service evaluation [7] and four letters to the editor reporting retrospective note reviews [8–11]. Two literature reviews [12,13] were retrieved (both appear to be scoping reviews although the review methodology was not stated).

#### Participants

Sample sizes in the retrieved empirical studies ranged from 8 to 25 participants involved in semistructured interviews [1,2,4,5] and 11 to 704 respondents in surveys [3,6,8]. Publications reporting retrospective note reviews included between 84 and 2000 records [6,8–11]. Ten publications reported participant characteristics: five studies included healthcare providers [1,3,6–8], three included patients [4–6], and two studies included carers or family members [2,8]. Five publications reported the outcome of retrospective reviews of deceased patients' records [6,8–11].

None of the retrieved evidence reported participant characteristics such as gender, ethnicity, or religion. Of the two literature reviews, one focused on how healthcare professionals could impact on the number of eye donations from their clinical areas, outlining the potential benefits and considerations when involving patients in eye donation discussions [12]. The second focused on involvement of patients and family members in eye donation discussions [13].

### Potential for eye donation

Findings from retrieved retrospective note reviews were conducted in one palliative care and four hospice care settings. Data from the hospice settings reported between 52/100 (52%) and 164/174 (94%) of deceased patients could potentially have been eye donors [6,8–10] and in the palliative care setting the potential was 229/704 (35.2%) [11].

In aggregating data from these retrospective reviews, the potential for eye donation from hospice care settings was 313 and from palliative care settings 229. This suggests a potential donor population of 542; however, these figures relate to patients who were assessed as medically suitable to be eye donors, but we cannot extrapolate how many patients would have agreed to eye donation if asked. However, limited evidence suggests that discussions about eye donation can increase donation rates [6–8].

#### Summary: Objective 1

Mapping of the global literature retrieved little evidence exploring barriers and facilitators to eye donation from palliative and hospice care settings was available before 2001 and that a limited range of study designs/evidence synthesis methodologies had been adopted in the reported empirical work. Nine of the 13 publications were conducted in the United Kingdom with a dearth of literature from other countries and cultures. With the United States and India reportedly supplying 55% of all corneas available globally,^1^ it is surprising that there is no literature from these countries.

Although the evidence available includes representation from relevant participant groups patients, family members, and healthcare providers, the sample sizes are frequently small; however, the themes generated by the retrieved publications speak to recurring barriers and facilitators. To date the available literature base is very slim with a lack of high-quality primary research adopting mixed methods of investigation/exploration that would support practice and policy development.

### Objective 2: To identify the factors that are evidenced as informing or influencing the discussion of eye donation in hospice and palliative care settings

Analysis for Objective 2 focused on identifying factors that were evidenced as informing or influencing the end-of-life option of eye donation in hospice and palliative care settings applying qualitative content analysis.^30^ Coding of articles was performed by B.C.M.-S. and reviewed by T.L.-S. after development of a coding handbook. Coding focused on identifying barriers and facilitators to the option of eye donation being discussed with patients and family members. Codes were grouped under two category headings: (1) Attitudes toward eye donation (with subcategories beliefs and perceptions), (2) Knowledge (with subcategories assets and deficits).

#### Evidenced attitudes toward eye donation (including beliefs and perceptions)

Attitudes is defined as a learned tendency to evaluate things people, issues, objects, or events in a certain way.^31^ Evaluations are often positive or negative and informed by a person's beliefs and perceptions.^31,32^ Findings in this section have been synthesized from four studies reporting the attitudes of healthcare providers [1,3,6,8], three reporting the attitudes of patients [4–6] and three reporting the attitudes of carers or family members [2,7,8].

Healthcare providers are reported to be generally favorable toward eye donation, perceiving it as worthwhile [1,3,6,8]. Authors report that although participants felt uncomfortable discussing eye donation, the majority felt it was their professional responsibility to do so [1]. Similarly, Gillon et al.[3] exploring attitudes, knowledge, practice, and experience of corneal donation across a sample of 410 HCPs respondents report that 70% (291/410) perceived corneal donation as a rewarding opportunity for patients and/or their families and 82% (345/410) reported that corneal donation was compatible with their personal beliefs [3].

Furthermore, survey findings [8] report that 42% (8/14) of doctors raised the issue of eye donation based on their experience that the option was perceived by patients and family members as a way of giving something back to society. Of note is that although HCPs acknowledge that eye donation is worthwhile, evidence indicates that discussing eye donation is not common practice [3,6,8,12]. Specifically, two surveys including HCPs found that 92% (92/100) and 93% (399/431) never or rarely raised the subject of corneal donation with patients or relatives [3,6].

Authors suggest that HCPs' perception that discussing eye donation will cause distress to patients and family members is a barrier to eye donation [1,3,8,12]. For example, retrieved publications reported that healthcare professionals believed that discussing eye donation would detract from the tranquil environment of a hospice and that donation requests could cause patients and their families physical and psychological harm [1,3]. However, service evaluation data reports that 86% (12/14) of doctors reported that conversations did not cause additional distress with 57% (8/14) reporting that the conversations about eye donation were perceived by patients and families as a positive outcome from the death [8].

Of note is that HCPs' perception that discussing eye donation would cause distress was not supported, in the retrieved records [4–8]. Three studies reported the attitudes, beliefs, or perceptions of patients [4–6], indicating that patients were willing to participate in discussions about the option of eye donation [5,6] but that patients were unaware of the option of eye donation or assumed that they were ineligible. Furthermore, participating patients were motivated to be eye donors and felt positive about the possibility of helping others [5,6].

A survey of inpatients [6] found that the majority of participants 73% (8/11) reported that they did not find it upsetting to discuss eye donation and that asking about donation enabled them to make an informed decision about donation. A further potentially important finding is that participants reported their preference to talk about eye donation while they were still well rather than when deteriorating [5].

Comments from nursing logs [7] after the introduction of an admission script that included questions about eye donation confirmed that patients (*n* = 121) and families were not aware of their eligibility to donate their eyes, but they were not concerned about the topic of eye donation being mentioned during admission. Nurses were positive about introducing the option of donation at admission [7].

Only one study mentioned cultural and religious beliefs as a barrier to eye donation [4], the study explored the views of 25 terminally ill cancer patients toward eye donation. The majority of patients [14/25 (56%)] were unwilling to donate their eyes based on their Buddhist beliefs that the body must remain untouched for eight hours after death to allow the spirit to depart and remain intact as the spirit should be able to see in the afterlife [4].

Publications that reported family/carer attitudes toward donation [2,8,12,13] found a lack of awareness of their dying family member's eligibility to be a potential eye donor. Findings indicated a range of beliefs including that donation was right, is a social duty to donate, and that it would be “wasteful” not to [2]. Family members' decision to decline eye donation was based on the prior stated wishes of the patient not to donate or the family's uncertainty about the patient's wishes [2,8].

Findings from across the retrieved dataset indicate that HCPs are a key barrier to the option of eye donation being raised [1,3,6,8] usually avoiding discussions about eye donation unless the issue is raised by the patient or the patient's family [1,3,6,12]. Although HCPs were cautious about discussing eye donation, patients [5,6] and carers [2,7,8] wanted to be informed and were not averse to holding discussions about eye donation. This points to a clear disconnect between the perceptions and beliefs of service providers and the perceptions of services users as reported in the existing literature.

## Knowledge: Assets and Deficits

Five publications included HCPs reports of their knowledge about eye donation [1,3,6–8]. In all studies HCPs reported knowledge deficits including not having sufficient knowledge about the process of eye donation [1] and lacking confidence to initiate eye donation discussions [3,6–8]. However, training is not a guarantee that eye donation would be discussed [3] A study in the United Kingdom reported that 115/433 (27%) of HCPs had received some information, education, and training on eye donation, but 399/431 (93%) rarely or never raised the option of eye donation, with 357/433 (83%) of HCPs reporting that they did not know enough about eye donation in general terms to discuss it with patients and their families [3].

Furthermore, reviews of the literature including HCPs confirm the facilitative impact of education and training and that willingness to discuss donation is positively correlated to knowledge about the process of eye donation (referral and retrieval) and being aware of local policy and guidance [12,13].

Key knowledge deficits synthesized from the retrieved publications indicate that hospice and palliative care patients are generally unaware about eye donation and eligibility criteria [4–6]. For example, in two studies, patients thought they could transmit their cancer to recipients [4,5] or that their eyes would not be good enough for use in transplantation [4]; furthermore, next of kin are unaware that their dying family member with cancer could donate their eyes [2]. Retrieved evidence further indicates that not knowing the beliefs/wishes of the deceased regarding eye donation is a key barrier to increasing eye donation [2,8].

## Discussion

Current literature in the donation and behavior change contexts continue to link attitudes toward a topic leading to a specific behavior taking place. For example, if people have a positive attitude toward donation generally, they will be willing to donate; however, authors report that attitudes alone are a poor indicator of behavior as the context within which an action takes place will cancel out favorable attitudes (Bracher et al., in press).^33^

Early research reports no linear causal relationship between knowledge, values, attitudes, willingness, and action related to donation behaviours^34^ with further modeling supporting the general finding that behavioral intention (or willingness) does not predict action.^35^ Therefore, relying on changes in attitudes toward eye donation alone is not the route to increasing eye donation as the context within which discussions about eye donation need to take place is key.

This context within which discussions around eye donation need to take place is that of death. Apart from living donation, all donation options cannot proceed until someone has died; therefore, raising a topic that so profoundly signals impending death may be why HCPs are reluctant to raise the option of eye donation. A further consideration in the reluctance to raise the issue with family members is that death not only denies the next-of-kin of a significant relationship, but also robs them of many of their usual coping mechanisms, imposing a sequence of events that leave family members feeling dispossessed of physical and psychological equilibrium^36^; therefore, HCPs may avoid what they perceive to be “distressing topics” due to concerns about the reactions of family members [7].

The retrieved evidence indicates that patients and family members are not averse to, nor distressed by, discussions around the option of eye donation, however, as with all end-of-life discussions timing is key. Evidence supports the benefits in “introducing” this issue at admission with this discussion being merely to assess donation status [7]. For example, a general discussion around being on the donor register and carrying a donor card. Adding eye donation to admission protocols would offer the opportunity to clarify potentially long-held plans to be a donor, which with the onset of a cancer diagnosis, would be limited to tissue and eye donation.

Furthermore, in raising this issue as part of “the usual” admission process, patients and family members are then able to discuss this option if they wish to and seek further information and guidance. As reported by the Organ Donation Taskforce^37^ making donation “usual” as opposed to “unusual” is essential if donation rates are to increase.^37^

However, early indications from a national study into the potential of eye donation from hospice and palliative care settings (*EDiPPPP*) is that both clinicians and the public are poorly informed about the need for eye donation, potential donor eligibility criteria, and the process of eye donation. It is essential that empirically informed interventions are developed that successfully raise public awareness and clinical confidence and competent in operationalizing the end-of-life option of eye donation.

## Conclusion

This scoping review has provided an up-to-date appraisal of the current potential, perceptions, and practice underpinning offering the option of eye donation to dying patients and their family members in palliative and hospice care context.

Studies included in this review from one palliative care [6] and four hospice care [8–11] settings report that a total of 542 patients could potentially have donated their eyes. This equates to >1000 eyes from just these settings that could make a significant contribution to sight saving and sight restoring treatment and surgery.

The review outlines the key barriers to increasing eye donation from these settings include the reluctance of HCPs to raise this issue to avoid causing perceived distress to patients and their next of kin, and the evidenced lack of awareness of patients and family members about their own or their relatives donation options and eligibility. This review also indicates a lack of clinical guidance drawn from high-quality evidence proposing interventions that could inform HCPs' practice. The absence of this guidance is a barrier to change.

### Limitations

- *Search strategy:* in many articles, “eye donation” was subsumed under a wider term such as “tissue donation” or “organ donation.” This has implications for search strategies in future reviews focusing on eye donation, as relevant terms many not be directly visible to searches with a resulting risk of excluding articles that include eye donation as a subset of wider investigations.
- MeSH headings did not reliably include all relevant studies relating to the focus of this search. Those developing search strategies to underpin reviews may find it helpful to use a test set of familiar articles, as we have done here.
- None of the retrieved records included a diverse cultural participant group nor specifically looked at variables such as age, gender, and religious views, despite religious/cultural factors being evidenced as factors that influence organ and tissue donation decision making.^38–40^

## Abbreviations Used

HCP: health care provider
JBI: Joanna Briggs Institute
ODR: Organ Donor Register
PCC: Population (P) Concept (C) and Clinical Context (C)
PRISMA-ScR: Preferred Reporting Items for Systematic Reviews and Meta-Analyses Extension for Scoping Reviews
